# Differentiation of benign and malignant superficial soft tissue lesions using real-time strain elastography

**DOI:** 10.3906/sag-2101-166

**Published:** 2021-08-14

**Authors:** Gökçe ANNAÇ, Murat CANYİĞİT, Sinan TAN, Ersin AKŞAM, Nuran SÜNGÜ ADIYAMAN, Halil ARSLAN

**Affiliations:** 1 Department of Radiology, Bartın State Hospital, Bartın, Turkey; 2 Department of Radiology, Ankara City Hospital, Ankara, Turkey; 3 Department of Radiology, Faculty of Medicine, Kırıkkale University, Kırıkkale, Turkey; 4 Department of Plastic, Reconstructive and Arsthetic Surgery, Faculty of Medicine, Katip Çelebi University, İzmir, Turkey; 5 Department of Pathology, Ankara City Hospital, Ankara, Turkey

**Keywords:** Benign, malignant, cutaneous, subcutaneous, real-time strain elastography

## Abstract

**Background/aim:**

To evaluate benign and malignant cutaneous-subcutaneous lesions using real-time strain elastography (RTSE) and to compare the findings with histopathologic results.

**Materials and methods:**

Over a period of 10 months, 72 patients (38 with benign and 34 with malignant cutaneous and subcutaneous lesions) were prospectively included in this study. Elasticity patterns and strain ratios were examined for each lesion. Lesions were evaluated in 4 groups as yellow-red (soft; pattern-1), green-yellow (moderate; pattern-2), blue-green (hard; pattern-3) and blue (hardest; pattern-4). The stiffness of the lesions was displayed with strain ratios by comparing of a nearby reference tissue. The recorded images were compared with histopathologic findings.

**Results:**

On sonoelastograms, considering patterns 1–2 as benign and patterns 3–4 as malignant, the sensitivity, specificity, and positive and negative predictive values for the differentiation of malignant from benign lesions were 100%, 68.5%, 74%, and 100%, respectively. Considering a cut-off value of the strain ratio as > 3.05, the sensitivity, specificity, and positive and negative predictive values were 91%, 89%, 88%, and 92%, respectively. The area under the curve (AUC: 0.972) showed the excellent ability of strain elastography to differentiate benign and malignant lesions.

**Conclusion:**

RTSE is an important imaging tool to differentiate benign and malignant superficial soft tissue lesions. Our results suggest that RTSE can be used to predict malignancy since malignant lesions are more confidentially diagnosed than benign superficial soft tissue lesions on elastograms.

## 1. Introduction

Nowadays, cutaneous-subcutaneous lesions are easily assessed with high-frequency linear probes. Although sonoelastography is not yet used in routine clinical practice, it has been shown previously that it is useful in the assessment of stiffness of tissues. There are several types of sonoelastography for the assessment of tissue elasticity, such as acoustic radiation force impulse (ARFI) and transient elastography (TE), which provide quantitative measurements. Shear wave elastography (SWE), which relies on the ARFI technique, uses shear waves generated by an internal and external mechanical push [1,2]. In this study, we used real-time strain elastography (RTSE), which is a sonography-based imaging modality that measures the elasticity of soft and hard tissues semiquantitatively under iterative compression force applied to the tissue surface [3].

Sonoelastography was first used experimentally in 1980 by Ophir et al. [4]. This was the first study to show that fibroadenomas proved to be eight times softer than breast cancer lesions by using sonographic grey-scale elastography. Since then, sonoelastography has been used increasingly, and successful results have been obtained in thyroid, breast, prostate, lymph nodes, and plantar fascia [5–11]. Assessment of superficial lesions using sonoelastography is easier than in deep lesions according to previous studies [12,13].

In general, cutaneous-subcutaneous lesions are assessed through palpation and removed surgically, which is invasive and uncomfortable for patients. Whereas, RTSE is safe and noninvasive; therefore, it may be helpful to distinguish benign and malignant lesions to avoid needless surgical attempts. The aim of the present study was to investigate the role of RTSE in differentiating benign and malignant cutaneous-subcutaneous lesions.

## 2. Materials and methods

### 1.1. Patients

**Figure 1:**
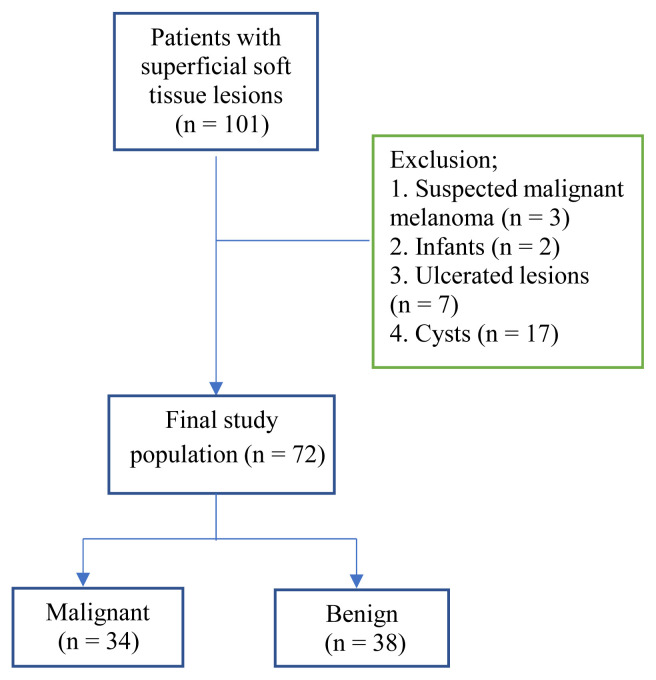
Flowchart of the study.

This observational prospective study was approved at our institution by the institutional review board. Over a 10-month period, 101 patients were referred for surgical excision of superficial soft tissue lesions and underwent sonographic examinations, including RTSE. Before enrollment, each patient gave written informed consent. The patients who were suspected of having malignant melanoma (n = 3) were not included in our study and sent to reference hospitals for sentinel lymph node biopsy. Because sentinel lymph node biopsy was not able to be performed in our hospital. The infants (n = 2) were excluded because there were difficulties with cooperation. We excluded ulcerated lesions (n = 7) because of the technical difficulty to perform sonoelastography for irregular surfaces. We excluded the anechoic cysts and the cysts including some degree of echogenic components, which have partial anechoic appeareance and posterior acoustic enhancement on sonographic examination (n = 17). Therefore, a final study cohort of 72 patients remained (Figure 1).

### 1.2. Real-time strain elastography (RTSE)

Sonoelastography was performed using a real-time strain imaging sonoelastography device (General Electrics Logiq E9), and a 6–15 MHz linear probe was used in all patients. The images were acquired in real time on the video screen. One radiologist (G.A.) who had 4 years of sonoelastography imaging experience examined all images. The ultrasound examination started with B-mode imaging and then continued with RTSE imaging. Both elastographic and B-mode images were presented as a two-panel image at the same time. A target lesion on the B-mode image was demonstrated on a color scale on elastograms. Multiple iterative compressions and decompressions to the tissue surface were performed by the transducer to obtain a better signal-noise ratio on elastograms until a stable image was obtained. The quality factor of compression applied to the lesions, represented on a bar scale of 1–7, was used to select the optimal image, and images that were acquired with adequate compressions (bar scale of 5–7) were evaluated.

**Figure 2:**
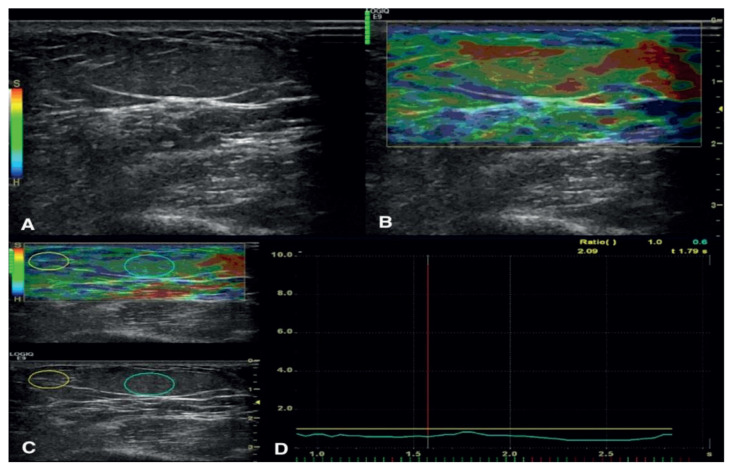
Lipoma located on the forearm of a 60-year-old woman is evaluated by B-mode US (a) and RTSE. The green-yellow lesion on strain elastography is grouped as type 2 lesion (b). The ratio of the lesion strain to the nearby subcutaneous tissue strain is calculated (c). The strain ratio yields 0.6 (d). The lesion was excised totally, and histopathology confirmed the diagnosis of lipoma.

**Figure 3:**
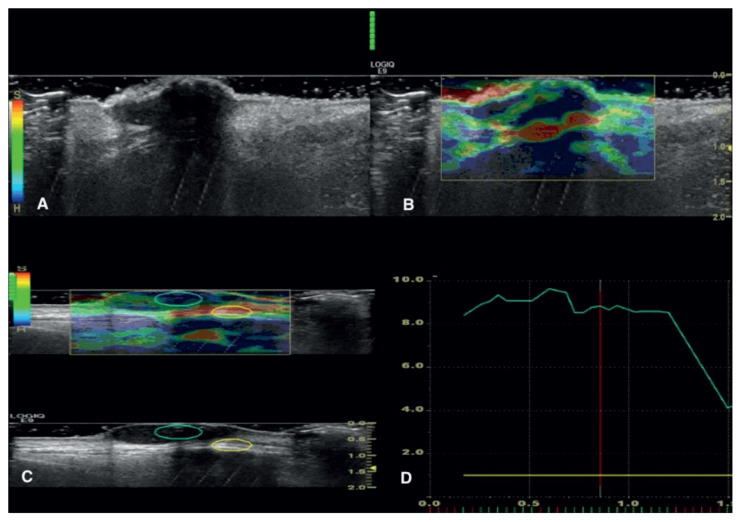
BCC lesion on the upper lip of an 83-year-old man is evaluated using B-mode US and RTSE. The hypoechoic lesion with indistinct border showed on B-mode (a) is grouped as type 4 lesion (blue, the hardest) according to the elasticity pattern (b). The ratio of the tumor strain to the subcutaneous tissue strain is calculated (c). The strain ratio is found as 8.8 (d). This also confirms the histopathologically verified malignancy.

All elastograms, static images, and video sequences were evaluated by a radiologist who was blinded to the histopathologic results. The stiffness of the lesions was displayed with strain ratios and a color overlay on elastograms. The strain ratios of the superficial lesions were calculated by comparing the adjacent tissue outside the lesion. The first region-of-interest (ROI) was placed on the adjacent tissue, the second ROI was placed on the lesion. The ROI was chosen as large as possible to include the entire lesion with the boundaries. The ratio of the ROIs gave the strain ratio, which was calculated automatically by the sonoelastography device (Figure 2a–2d, Figure 3a–3d).

### 1.3. Image interpretation

**Figure 4:**
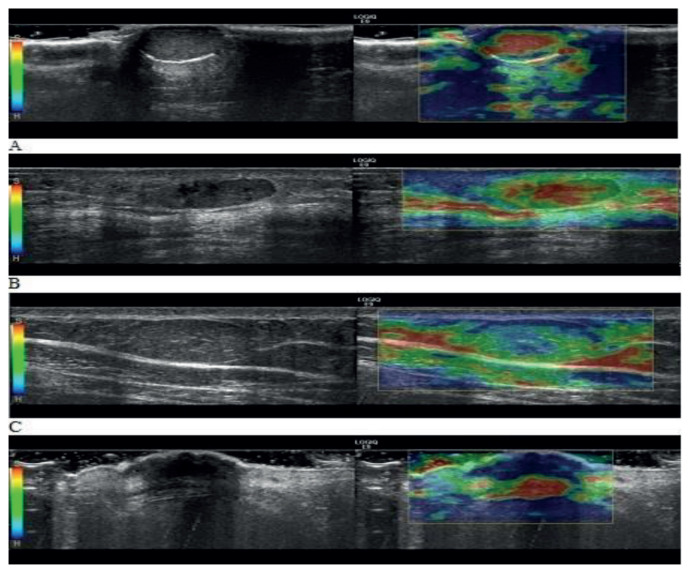
Elasticity patterns according to the color scale: pattern 1 (a), pattern 2 (b), pattern 3 (c), pattern 4 (d).

For each patient, elasticity patterns and strain ratios were assessed on elastograms. The images were scored for elasticity patterns according to a scoring system proposed by us. All lesions were divided into 4 groups according to the color scale as follows (Figure 4a–4d):

Pattern 1 (soft): The lesion was red-yellow.
Pattern 2 (hard): The lesion was almost green.
Pattern 3 (harder): The lesion was green-blue.
Pattern 4 (hardest): The lesion was almost blue.

### 1.4. Reference standard

All 72 lesions were totally excised surgically after real-time strain elastography imaging. Histopathologic findings were considered as the reference standard for each lesion.

### 1.5. Statistical analyses

Statistical analyses were performed using a statistical software package (Statistical Package for the Social Sciences version 15.0 Chicago, IL, USA). Quantitative data are presented as means ± standard deviation (SD).

The lesions were classified into two groups as benign and malignant. The quantitative variables of the groups were compared using the independent t-test, Fisher’s exact test, and the chi-square test. The diagnostic value (sensitivity and specificity) for elasticity patterns and strain ratios were assessed by exploring the receiver operator characteristics (ROC) curve. The area under the curve (AUC) demonstrated the capability of real-time strain elastography statistically in the differentiation of benign and malignant lesions.

The diagnostic value for the mean age difference between women and men were assessed using the independent samples t-test. The chi-square test was used to assess differences in the elastographic patterns of benign and malignant lesions, as well as differences in sex between the groups. P < 0.05 was considered to indicate significance.

## 2. Results

**Figure 5:**
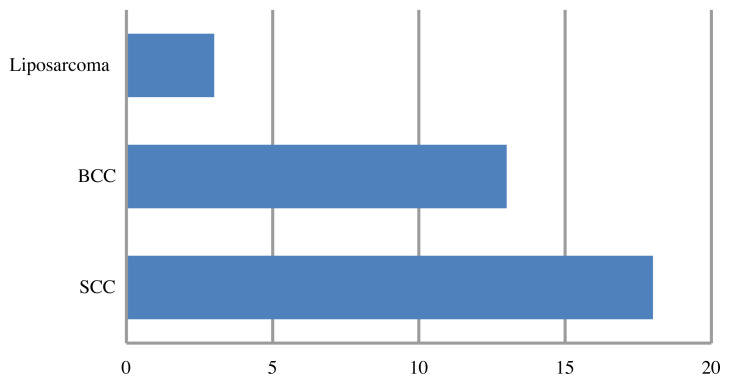
In the malignant category, there were 18 squamous cell carcinomas (SCC), 13 basal cell carcinomas (BCC), and 3 liposarcomas.

**Figure 6:**
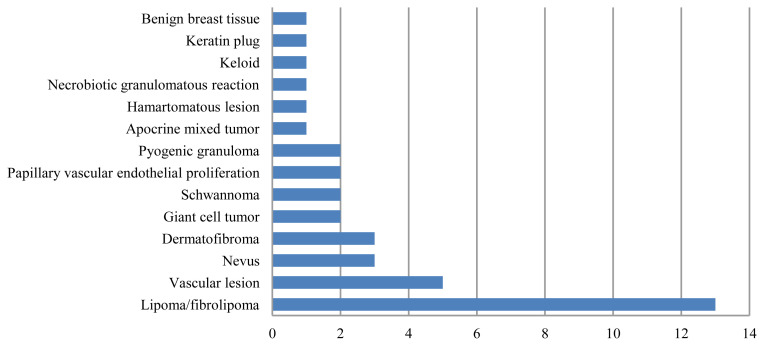
The benign lesions comprised 9 lipomas, 4 fibrolipomas, 5 vascular lesions (venous/cavernous hemangioma, arteriovenous malformation), 3 nevi (compound, intradermal), 3 dermatofibromas, 2 giant cell tumors, 2 schwannomas, 2 papillary vascular endothelial proliferations, 2 pyogenic granulomas, 1 apocrine mixed tumor, 1 hamartomatous lesion, 1 necrobiotic granulomatous reaction, 1 keloid, 1 keratin plug, and 1 benign breast tissue.

Histopathological evaluations yielded 34 malignant and 38 benign lesions out of 72 patients. The histopathological results of the lesions are presented in Figures 5 and 6. The most common malignant lesions were squamous cell carcinomas (SCC) (n = 18, 52%) and basal cell carcinomas (BCC) (n = 13, 38%), whereas the most common benign lesions were fibro/lipomas (n = 13, 34%) and vascular lesions (venous/cavernous hemangioma, arteriovenous malformation) (n = 5, 13%).

There was a statistically significant difference between the elasticity patterns of benign and malignant lesions (p < 0.001); 26.3% of the benign lesions were pattern 1, 42.1% were pattern 2, 23.7% were pattern 3, and 7.9% were pattern 4. In the malignant group, 41.2% of the lesions were pattern 3, and 58.8% were pattern 4; however, there were no pattern 1 or 2 (Table 1). Patterns 1 and 2 were considered as benign, pattern 3 was probably malignant, and pattern 4 was malignant. The sensitivity of the detection rate of malignant lesions was 100%, specificity was 68.5%, and the positive and negative predictive values were 74% and 100%, respectively in qualitative analyses.

There was a statistically significant difference between the strain ratios of benign and malignant lesions (p < 0.001). The average strain ratio of the benign lesions was 1.62, whereas it was 5.48 in malignant lesions (Table 2).

The strain ratio scores of benign and malignant lesions were classified into 4 groups as 0–2, 2–4, 4–6, and > 6, and there was a statistically significant difference between the groups of strain ratios (p < 0.001). The strain ratio for 71% of benign lesions was between 0–2, and there was no benign lesion with a strain ratio > 6. The strain ratio for 50% of malignant lesions was between 4–6, and > 6 for 38.2% of malignant lesions. No malignant lesions had a strain ratio between 0–2 (Table 2).

The diagnostic performance of the quantitative measurements of benign and malignant lesions were evaluated using ROC curves. In ROC analyses, if the cut-off value for the strain ratio was 3.05, the sensitivity of the detection rate of malignant lesions was 91%, specificity was 89%, and the positive and negative predictive values were 88% and 92%, respectively, for values greater than 3.05. The AUC was calculated as 0.972.

The mean age of the 72 patients was 51.1 ± 20.3 (range, 3–87) years. The mean age of the women was 43.2 ± 21.4 years, whereas it was 57.8 ± 17 years for men. In the malignant group, the mean age was 64.6 ± 14.6 years, whereas it was 39.02 ± 16.9 years in the benign group. There was a statistically significant difference between the mean age distributions of the benign and malignant lesions (p < 0.0001). Sixteen (42.1%) of 38 benign lesions were in men, whereas 23 (67.6%) of 34 malignant lesions were in men. The p value of Z-test for the difference between proportions of having malignancy in both sexes was found 0.044. The ratio of men in the malignant group was significantly higher than in the benign group. The lesions were located in different parts of the body. All basal cell and squamous cell carcinomas were located in the facial region, and all liposarcomas were located on the extremities. Other lesions were located in various parts of the body such as the extremities, abdominal surface, chest, and face. The size of the lesions ranged from 8 mm to 5 cm. Demographics of the study population are presented in Table 3.

## 3. Discussion

This study showed that quantitative and qualitative RTSE parameters are useful for malignancy prediction of superficial soft tissue lesions. Strain ratio can be used to differentiate malignant lesions from benign lesions. According to our results, the sensitivity and the specificity of the detection rate of malignant lesions was around 90% if the cut-off value for the strain ratio was 3.05. Elasticity patterns were also reliable for malignant lesions, whereas there was substantial overlap for the elasticity patterns of benign lesions.

In the present study, elasticity patterns and strain ratios were evaluated to differentiate benign and malignant lesions. Different classifications were used for elasticity patterns in various studies. We used four types of elasticity patterns for the simple and efficient classification of superficial lesions. According to the elasticity patterns, all malignant lesions had pattern 3 or pattern 4, and approximately 70% of benign lesions had pattern 1 or pattern 2 on elastograms. In other words, the blue color represented malignancy, and the red color represented benign tissue, consistent with the previous studies [14–16]. Despite the statistically significant difference between the elasticity patterns of benign and malignant lesions, there was a substantial overlap, because more than 30% of benign lesions had a malignant pattern. From this aspect, the accuracy of the elasticity pattern, which is a subjective method, is suspect. Despite the high sensitivity rates in the detection of malignancy, stand-alone elasticity pattern evaluations may lead to misinterpretation of lesions. Therefore, careful evaluation of elasticity patterns by experienced observers on elastographic color images is required.

We analyzed the strain ratio, which is a more objective method than the elasticity pattern. The strain ratio is a quantitative measurement of the hardness of a lesion in respect to the adjacent soft tissues. We chose a cut-off value of 3.05 for the differentiation of malignant and benign lesions, which was closer to the mean strain ratio of the benign group than the mean strain ratio of the malignant group. In our study, the area under the ROC curve (AUC = 0.972) showed the excellent ability of RTSE in the differentiation of benign and malignant lesions. Unlike our study, the study of Tavare et al., shear-wave elastography of benign and malignant musculoskeletal lesions, reported that a single cut-off value was not chosen due to altered accuracy by lesion position and patient age [17]. In our study, qualitative and quantitative analyses by cut-off value showed that there was a good correlation between strain elastography and histopathologic findings. Dasgeb et al. found similar results to our study that all benign lesions had strain ratios ≤ 3.0, whereas all malignant lesions had strain ratios ≥ 3.9 [18]. In a study with SWE [19], the sensitivity of the detection rate of malignant soft tissue lesions was 91.9%, specificity was 72.2%, whereas, in our study, the sensitivity was 91%, specificity was 89%. Quantitative analysis demonstrated that specificity in the diagnosis of malignant lesions was higher in our study.

Only the study of Zaitsev et al., a pictorial essay on the elastographic evaluation of soft tissue tumors, reported that sonoelastography did not lead to any benefit as a stand-alone diagnostic technique except with diffuse lipomas, fibrolipomas, desmoids, and low-grade liposarcomas [20]. In our study, 3 liposarcomas and 13 fibrolipomas/lipomas were evaluated. Two of the 3 liposarcomas were displayed as pattern 3, and the strain ratios were 3.5 and 4. The other liposarcoma was displayed as pattern 4, and the strain ratio was 4.5. Although liposarcomas demonstrated high strain ratios and elasticity patterns, none of the lipomas/fibrolipomas had elasticity patterns greater than 2. Generally, the strain ratios of lipomas were similar to the subcutaneous lipomatous tissue laying near them. The strain ratios of lipomas and fibrolipomas ranged between 0.5–2, which is consistent with the study of Lee et al [21].

In our study, keratin plug, keloid, and necrobiotic granulomatous reactions had high strain ratios, 3.8, 4.5, and 5, respectively, as with malignant lesions. Although they were benign lesions, the high strain ratios might be explained by the presence of scar and fibrous tissue. The study of Friedrich-Rust et al., in which RTSE was used for the noninvasive assessment of liver fibrosis in chronic viral hepatitis, demonstrated that there was a high correlation between histologic liver fibrosis stages and elastograms, with increasing elasticity scores [22]. In another study by Ferraioli et al., it was mentioned that the liver fibrosis index in RTSE was in direct correlation with the fibrosis score of the liver [23]. The fibrous tissue had high strain ratios due to being a hard tissue, consistent with these studies. Therefore, the assessment of benign lesions, including fibrous tissue, should be performed carefully so as not to misdiagnose benign tissue as malignant lesion.

In our study, we excluded patients with cystic lesions because most cysts were represented with a blue color on elastograms and did not exhibit significant strain ratios to exclude malignancy. In principle, if the medium is composed of pure liquid, the compression force on the medium will not produce significant strain ratios because the compression force is not transferred to pure water. However, most cysts have some degree of elasticity, suggesting the semisolid or viscous content of the cyst. A study by Patel et al. demonstrated that all examined testicular epidermoid cysts exhibited blue color on elastograms, as in our study [24]. However, in a study of Yeoh et al., epidermoid cysts were found to have higher shear modulus compared with other cystic lesions on SWEs, which means cystic lesions may have some degree of elasticity [25]. In another aspect, Bhatia et al. and Lyshchik et al. emphasized that sonoelastography could be used for the prediction of malignancy but only if cystic thyroid nodules were excluded, similar to our study [26,27].

Our study has some limitations. First, RTSE is a highly operator-dependent technique, and strain ratios may show variations due to the applied force on the tissue surface. Secondly, there was no interobserver comparison of the images in our study because of the lack of an experienced radiologist in performing RTSE in our department. A limited number of lesions that had fibrous tissue were also included in our study. Further studies with a larger number of cases should be performed to distinguish malignant lesions from benign lesions including fibrotic tissue. Another limitation of our study is the small size of the cutaneous lesions, which caused difficulty in the assessment of these lesions. Although we used a thick gel layer for superficial lesions, we should use a gel pad to provide better visualization of near-field areas. Despite this, we do not believe that it would change the results of the study.

In conclusion, we demonstrated that RTSE could be used as a noninvasive diagnostic technique to evaluate benign and malignant cutaneous-subcutaneous lesions. Although benign lesions may show false positive results and mistaken for a malignant lesion, the qualitative and quantitative findings of RTSE are more confidential in malignancy prediction of the superficial soft tissue lesions.

## Figures and Tables

**Table 1:** Distribution of benign and malignant lesions according to elasticity patterns.

Group	Elasticity pattern				Total
	Yellow-red	Green	Blue-green	Blue	
**Benign**	10 (26.3%)	16 (42.1%)	9 (23.7%)	3 (7.9%)	38
**Malignant**	0 (.0%)	0 (.0%)	14 (41.2%)	20 (58.8%)	34
**Total**	10 (13.9%)	16 (22.2%)	23 (31.9%)	23 (31.9%)	72

**Table 2:** Distribution of benign and malignant lesions according to groups of strain ratios.

Group	Groups of strain ratios				Total
	0–2	2–4	4–6	> 6	
**Benign**	27 (71.0%)	8 (21.0%)	3 (7.9%)	0 (.0%)	38
**Malignant**	0 (.0%)	4 (11.8%)	17 (50.0%)	13 (38.2%)	34
**Total**	27 (37.5%)	12 (16.7%)	20 (27.8%)	13 (18.0%)	72

**Table 3:** Demographic data of the study.

*Variable*	*Total ( n= 72)*	*Benign (n = 38)*	*Malignant (n = 34)*

**Age**	51.1 ± 20.3	39 ± 16.9	64.6 ± 14.6
	range, 3–87	range, 3–67	range, 21–87

**Sex**			
**Male**	39 (54.1%)	16 (42.1%)	23 (67.6%)
**Female**	33 (45.8%)	22 (57.8%)	11 (32.3%)

**Mean diameter (mm)**	38	36	32
	range, 8–50	range, 12–50	range, 8–42

**Localisation**			
**Head-neck**	39 (54.1%)	8 (21%)	31 (98%)
**Trunk**	11 (15.2%)	11 (28.9%)	-
**Abdomen**	9 (12.5%)	9 (23.6%)	-
**Upper extremity**	6 (8.3%)	4 (10.5%)	2 (5.8%)
**Lower extremity**	7 (9.7%)	6 (15.7%)	1 (2.9%)
